# The incidence and risk factors of deep venous thrombosis in lower extremities following surgically treated femoral shaft fracture: a retrospective case-control study

**DOI:** 10.1186/s13018-021-02595-z

**Published:** 2021-07-09

**Authors:** Zhixin Ren, Yufei Yuan, Wei Qi, Yanbao Li, Pengcheng Wang

**Affiliations:** 1 Department of Orthopaedic Surgery, The 3rd Hospital of Hebei Medical University, No. 139 Ziqiang Road, Shijiazhuang, 050051 Hebei People’s Republic of China; 2 Department of Orthopaedic Surgery, Handan Central Hospital, Handan, 056000 Hebei People’s Republic of China; 3 Department of Orthopaedic Surgery, The First People’s Hospital of Taian, Tai’an, 271000 Shandong People’s Republic of China

**Keywords:** Postoperative deep venous thrombosis, Femoral shaft fractures, Risk factors, Thromboprophylaxis

## Abstract

**Background:**

There is still a lack of data on deep vein thrombosis (DVT) following surgically treated femoral shaft fracture (FSF). The goal of this study was to investigate the characteristics of postoperative DVT and the association between the occurrence of DVT and risk factors in patients undergoing surgical treatment for FSF.

**Methods:**

This observational retrospective case-control study reviewed 308 patients who received surgical treatment of FSF between January 2016 and October 2020 at a university hospital. Univariate analyses were performed on the data of demographics, comorbidities, laboratory biomarkers, and operation-related indexes. The receiver operating characteristic (ROC) curve analysis, univariate analyses, and multivariate logistic regression analysis were employed to identify the independent risk factors associated with DVT.

**Results:**

In total, 308 patients with surgically treated FSF were included, among whom 48 (15.6%) patients had postoperative DVTs. The univariate analyses showing significant differences regarding DVT were American Society of Anesthesiologists (ASA) classification, diabetes mellitus, current smoking, aspartate transaminase (AST), and very-low-density lipoprotein (VLDL) level among the 34 factors. According to the ROC results, the optimal cutoff values for intraoperative blood loss, d-dimer, and age were 350 ml, 1.08 μg/ml, and 35 years, respectively. The multivariable model demonstrated 4 significantly independent associations with postoperative DVT, including current smoking, intraoperative blood loss (> 550 ml), age (> 35 years), and d-dimer > 1.09 μg/ml.

**Conclusion:**

These risk factors as screening tools contribute to risk stratification of the occurrence of thromboembolic events. In addition, our findings would help orthopedic surgeons make a cross-specialty decision and implement targeted precaution measures for patients with FSF.

## Background

As is well demonstrated, deep venous thrombosis (DVT) contributes to principal sources of morbidity, pulmonary embolism, and even mortality in all-cause hospitalized patients, particularly in trauma centers. Femoral shaft fractures (FSFs), accounting for 24.2% of femoral fractures and 2.9% of total body fractures, are commonly seen in long bone injuries [1]. The hypercoagulability of the blood, venous stasis, and vascular endothelial injury are widely acknowledged as pathological conditions to the presence of DVT in lower extremities. In trauma settings, risk factors for the formation of DVT have been extensively reported including multiple systemic injuries, advanced age, immobilization, post-injury systemic inflammatory response, and inappropriate use of prophylactic and major orthopedic trauma (spinal injury, pelvic and hip fracture, etc.) [2–5]. These findings shed some light on the epidemiologic information that was applicable in certain scope but could hardly generalize to various populations.

A secular trend suggested a fivefold increase of hospitalized venous thromboembolic events during the past decade in China, and 7292 patients were diagnosed with DVT after surgery per 100,000 population in 2016 [6]. As the morbidity and risk factors of DVT following surgery remarkably vary in different research time points, regions, and fracture sites [7, 8], more attention should be paid to the postoperative DVT in FSF patients. Although the prevention and treatment of DVT have been intensively studied in patients sustaining major orthopedic surgery (hip fracture surgery, total hip or knee arthroplasty) [5, 9], there remains limited information on the clinical characteristics and risk factors of DVT in patients who underwent surgeries for FSF. Previous researches generally integrated FSF with other fractures in the lower limb distal to the hip but failed to separate the FSF for analysis [10–12]. As a result, the power of the evidence was compromised inevitably, due to the relatively small sample size, heterogeneity from different medical facilities, and various confounding covariables. Therefore, it is required to elucidate the contribution of isolated FSF to the first 3-month DVT following internal fixation.

By far as we know, few reports exist with strong evidence on incidence and risk factors of DVT within the first 3 months of osteosynthesis for isolated FSFs. Since all patients with FSFs at our institution received DVT screening of the bilateral lower extremities after surgery, we designed this retrospective study to investigate the prevalence of DVT and to explore the risk factors associated with DVT within 3 months after surgery for isolated FSF.

## Materials and methods

This retrospective case-control study was approved by the ethics committee of the Third Hospital of Hebei Medical University, and the requirement to obtain informed consent is waived due to the anonymous nature of the data. All the patients included in this study underwent surgical treatment for FSF in the Third Hospital of Hebei Medical University between January 2016 and October 2020. Our institution is a tertiary, referral, teaching, and the largest orthopedics specialty hospital in Hebei province, and in this hospital, about 30 thousand orthopedics surgeries are conducted each year, which provides treatment for a large number of uncommon and referral trauma cases. The surgical procedures were all performed under sterile conditions and general/spinal anesthesia. Patients were kept partial weight-bearing after fixation of FSFs for 6–12 weeks until callus formation was observed on radiographs. Hospitalized surveillance records and post-discharge follow-up records were used to identify the occurrence of DVT within the first 3 months after surgery. Postoperative blood tests, regular duplex ultrasonography (DUS) screening, and thromboprophylaxis therapy were conducted according to our institutional protocol. All the baseline characteristics and DVT results were extracted from the electronic medical record system. Standardized protocols for medical chart review were used by training study personnel who were blind to the diagnosis results of DVT and the type of surgery performed. A patient-reported history of comorbidities was confirmed with physical exam findings and an operative report.

### Inclusion and exclusion

The inclusion criteria were patients aged 18 years or older, definitive diagnosis of FSF, surgical treatment (osteosynthesis) received, and chemical thromboprophylaxis received after admission. The exclusion criteria were incomplete data, pathological (metastatic) or old (> 21 days since occurrence) fractures, open fractures, concurrent fracture in other locations, a history of venous thromboembolism, active malignancy, presence of hypercoagulopathy or hematological disorders, recent use of anticoagulants or oral contraceptives within 3 months, or preoperative diagnosis of DVT.

### DVT detection and prophylaxis

DVT was diagnosed by DUS according to the guideline for diagnosis and treatment of DVT updated by the Chinese Medical Association [13]. Although no strong evidence on the routine care of DUS was proposed by the American College of Chest Physicians Evidence-Based Clinical Practice Guidelines, our institution recommended DUS scanning to detect DVT as standard care for patients with FSF. Experienced ultrasonography radiologists who were blind to any laboratory results conducted the scanning on bilateral lower extremities before and after surgery, every 7 days postoperatively, before discharge, or when any signs and symptoms suggestive of DVT presented. DVT was confirmed based on the detection of venous lumen obstruction or filling defect in the common femoral vein, deep femoral vein, femoral vein, popliteal vein, posterior tibial vein, anterior tibial vein, or peroneal vein. Superficial and intramuscular venous thrombosis was not included in this study due to little clinical insignificance [14, 15].

A thromboprophylaxis regimen was performed for each patient before and after surgery, consisting of intermittent pneumonic compression (IPC) and chemoprophylaxis. The IPC was stopped once DVT was diagnosed. Prophylactic low-molecular-weight heparin (LMWH, 4100 IU, once daily) was given within the first 24 h after admission and withheld 12 h before surgery. Postoperative prophylactic drugs were resumed at 12 h after surgery for patients without DVT. Anticoagulation therapy (LMWH, 4100 IU, every 12 h) was prescribed to patients with DVT during the hospital stay, followed by rivaroxaban for 3 months.

### Data acquisition and factors of interest

Electronic medical records and operation reports were inquired for comorbidities and demographic data, consisting of sex, living area, age, body mass index (BMI), diabetes mellitus, hypertension, arrhythmia, chronic pulmonary disease, liver disease, current smoking, alcohol consumption, preoperative stay, total hospital stay, ASA classification (I–IV), anesthesia type (spinal vs. general), osteosynthesis [intramedullary nailing vs. open reduction internal fixation (ORIF) with plate and screws], intraoperative blood loss, the volume of intraoperative transfusion, and surgical duration. The BMI (kg/m^2^) was divided using the criteria recommended by the Chinese Working Group on Obesity: normal (18.5–23.9), underweight (< 18.5), overweight (24.0–27.9), and obese (≥ 28.0).

Blood samples were drawn on the first postoperative day. The results of hematological indices included total protein (TP) level, albumin (ALB) level, alanine transaminase (ALT), aspartate transaminase (AST), high-density lipoprotein cholesterol (HDL-C) level, low-density lipoprotein cholesterol (LDL-C) level, very-low-density lipoprotein (VLDL) level, serum sodium concentration (Na^+^), white blood cell count (WBC), neutrophil count (NEU), lymphocyte count (LYM), red blood cell count (RBC), hemoglobin (HGB) level, platelet (PLT), and d-dimer level.

### Statistical analysis

SPSS26.0 was used to perform all the statistical analyses (IBM, Armonk, NY, USA). Continuous data were examined by the Kolmogorov-Smirnov test, followed by Student’s t-test for variables with normal distribution and Mann-Whitney *U* test for those with the non-normally distribution. The variables were described as mean ± SD/median with quartile. Categorical data were evaluated by the chi-square or Fisher’s exact test, as appropriate, expressed as number and percentage (%). Continuous variables with *P* < 0.10 were subjected to receiver operating characteristic (ROC) analysis to determine the optimal cutoff values. Before entering logistic regression, the continuous variables were converted into categorical ones according to these cutoff values. All the categorical variables with *P* < 0.10 were entered into the multivariate logistics regression model to identify the independent predictors of DVT, and the correlation strength was indicated by odds ratio (OR) and 95% confidence interval (95% CI). *P* values less than 0.05 were regarded as statistically significant. The Hosmer-Lemeshow test was applied to assess the fitness of the final model, and a *P* value less than 0.05 was an acceptable result.

## Results

In our study, the analyses of overall 308 patients with FSF were conducted, comprising 211 males and 97 females, with an average of 44.5 years (SD, 18.7; range, 18–99; median, 41). The total hospitalization stay was 22.2 days on average (SD, 25.4; median, 16; range, 2 to 341). In the total 48 patients with DVT, 41 (85.4%) were diagnosed within 14 days after surgery. The average interval between operation and DVT diagnosis was 8.1 days (median, 6 days), ranging from 1 to 29 days.

Of the 308 patients who underwent FSF surgery, 48 had postoperative DVTs, indicating a 15.6% incidence of DVT. Thirteen (27.1%) patients had proximal veins involved, and 35 (72.9%) presented in distal veins. A total of 84 clots were observed by the DUS screening, with 1.75 (range, 1 to 5) thrombi for each patient on average. To be specific, 39 clots were detected in the peroneal vein, 27 in the anterior/posterior tibial vein, 10 in the popliteal vein, 5 in the femoral vein, 1 in the deep femoral vein, and 2 in the common femoral vein. There were 42 cases of DVT in the unilateral lower extremity and 6 in the bilateral lower limbs. To be noted, there were 11 (22.9%) DVT s appearing in uninjured extremities (see Table 1).

**Table 1:** The characteristics and locations of DVT presented in patients included in the study

Location of the postoperative DVTs	No. (%) of thrombi (***n*** = 84)	No. (%) of patients (***n*** = 48)
**Proximal DVTs**	18 (21.4%)	13 (27.1%)
Common femoral vein	2 (2.4%)	
Deep femoral vein	1 (1.2%)	
Femoral vein	5 (6.0%)	
Popliteal vein	10 (11.9%)	
**Distal DVTs**	66 (78.6%)	35 (72.9%)
Posterior tibial vein	26 (31.0%)	
Anterior tibial vein	1 (1.2%)	
Peroneal vein	39 (46.4%)	

Of the total 48 DVT-positive patients, none of them developed pulmonary embolism within the first 3 months after surgery. In addition, 21 (43.8%) DVTs achieved complete recanalization at a mean of 12.8 days after the first diagnosis, and 5 (10.4%) were partially recanalized. However, 22 (45.8%) patients showed neither propagation nor recanalization during the post-discharge period.

**Fig. 1:**
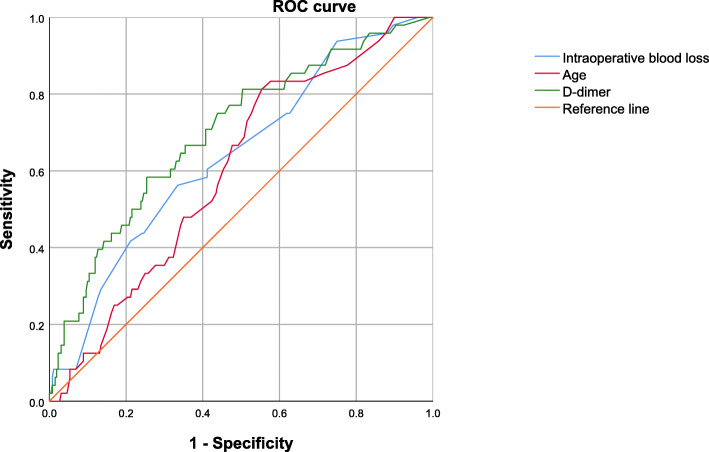
The ROC curve of three continuous variables with statistical significance cutoff values. The optimal predictive values of intraoperative blood loss, d-dimer, and age were 550 ml, 1.09 μg/ml, and 35 years, respectively

In the univariate analyses, there was no statistical significance between the two groups concerning ASA classification, anesthesia type, osteosynthesis type, the volume of intraoperative blood loss, or surgical duration (Table 2). Continuous variables with statistical significance (*p* < 0.10) were analyzed by using the ROC curve, and the results indicated the optimal cutoff values for intraoperative blood loss, d-dimer and age were 350 ml, 1.08 μg/ml, and 35 years, respectively. After categorizing by the cutoff values, intraoperative blood loss (*p* = 0.002), d-dimer (*p <* 0.001), and age (*p* = 0.024) revealed statistical significance with DVT (see Fig. 1, Table 3).

**Table 2:** Univariate analyses of the risk factors associated with postoperative DVT following femoral shaft fracture

Variables	No. (%) of DVT (***n*** = 48)	No. (%) of non-DVT (***n*** = 260)	***P***
**Sex (male)**	37 (77.1)	174 (66.9)	0.164
**Living area**			0.493
Rural	37 (77.1)	88 (72.3)	
Urban	11 (22.9)	72 (27.7)	
**Age**	48.94 ± 16.7	43.70 ± 19.0	0.075
> 35 years	39 (81.3)	144 (55.4)	0.001^#^
**BMI (kg/m**^**2**^**)**			0.486
< 18.5	1 (2.1)	10 (3.8)	
18.5–23.9	14 (29.2)	98 (37.7)	
24.0–27.9	23 (47.9)	115 (44.2)	
≥ 28.0	10 (20.8)	37 (14.2)	
**Diabetes mellitus**	8 (16.7)	21 (8.1)	0.061
**Hypertension**	9 (18.8)	60 (23.1)	0.509
**Arrhythmia**	1 (2.1)	6 (2.3)	0.924
**Chronic pulmonary disease**	1 (2.1)	1 (0.4)	0.178
**Liver disease**	2 (4.2)	6 (2.3)	0.457
**Current smoking**	10 (20.8)	31 (11.9)	0.095
**Alcohol consumption**	7 (14.6)	28 (10.8)	0.444
**Preoperative stay, days**	6.38 ± 5.2	6.88 ± 6.3	0.597
**Total hospital stay, days**	26.19 ± 15.0	21.54 ± 26.8	0.254
**ASA classification**			0.115
I	3 (6.3)	34 (13.1)	
II	27 (56.3)	168 (64.6)	
III	16 (33.3)	50 (19.2)	
IV	2 (4.2)	8 (3.1)	
**Anesthesia (general)**	34 (70.8)	155 (59.6)	0.143
**Type of osteosynthesis**			0.245
Intramedullary nailing	32 (66.7)	150 (57.7)	
ORIF with plate and screws	16 (33.3)	110 (42.3)	
**Intra-op blood loss, ml**	700.00 ± 471.4	495.54 ± 350.1	0.006
> 550 ml	27 (56.3)	87 (33.5)	0.003^#^
**Volume of intra-op transfusion**	424.96 ± 634.4	360.8 ± 501.0	0.197
**Surgical duration, min**	194.79 ± 76.1	181.87 ± 81.1	0.307
**TP (< 60 g/l)**	29 (60.4)	153 (58.8)	0.839
**ALB (< 35 g/l)**	32 (66.7)	145 (55.8)	0.161
**ALT (> upper limit)**	15 (31.3)	74 (28.5)	0.695
**AST (> upper limit)**	10 (20.8)	91 (35.0)	0.055
**HDL-C (< 1.1 mmol/l)**	72 (56.3)	147 (56.5)	0.970
**LDL-C (> 3.37 mmol/l)**	4 (8.3)	24 (9.2)	0.842
**VLDL (> 0.78 mmol/l)**	10 (20.8)	30 (11.5)	0.078
**Na+ (< 135 mmol/l)**	13 (27.1)	72 (27.7)	0.931
**WBC (> 10 × 10**^**9**^**/l)**	16 (33.3)	106 (40.8)	0.333
**NEU (> 6.3 × 10**^**9**^**/l)**	29 (60.4)	165 (63.5)	0.688
**LYM (< 1.1 × 10**^**9**^**/l)**	21 (43.8)	113 (43.5)	0.970
**RBC < lower limit**	42 (87.5)	207 (79.6)	0.202
**HGB < lower limit**	42 (87.5)	201 (77.3)	0.112
**PLT (> 350 × 10**^**9**^**/l)**	10 (20.8)	51 (19.6)	0.846
**d**-**dimer**	2.10 ± 2.1	1.02 ± 1.3	0.001
> 1.09 μg/ml	28 (58.3)	66 (25.4)	< 0.001^#^

*Note*: ALT, reference range: female, 7–40 U/l; male, 9–50 U/l. AST, reference range: female, 13–35 U/l; male, 15–40 U/l. RBC, reference range: female, 3.5–5.0 × 10^12^/l; males, 4.0–5.5 × 10^12^/l. HGB, reference range: females, 110–150 g/l; males, 120–160 g/l

^#^ROC analysis results

**Table 3:** The ROC curve analysis of continuous variables with statistical significance

Variable	Cutoff value	Sensitivity	Specificity	AUC (95% CI)	***P*** value
**Intra-op blood loss**	550 ml	56.3%	66.5%	0.640 (0.557–0.723)	0.002
**d**-**dimer**	1.09 μg/ml	58.3%	74.6%	0.700 (0.619–0.782)	< 0.001
**Age**	35 years	81.3%	44.6%	0.602 (0.523–0.681)	0.024

Together with the abovementioned factors, other variables including diabetes mellitus (*p* = 0.061), current smoking (*p* = 0.095), AST (*p* = 0.055), and VLDL (*p* = 0.078) were retained in the final model. Finally, 4 variables demonstrated significantly independent association with postoperative DVT, which were current smoking (OR = 2.902, *p* = 0.027), intraoperative blood loss (> 550 ml) (OR = 2.202, *p* = 0.043), age (> 35 years) (OR = 2.789, *p* = 0.017), and d-dimer > 1.09 μg/ml (OR = 4.562, *p* < 0.001) (Table 4). The Homser-Lemeshow test showed good fitness of the final model (X^2^ = 10.800, *p* = 0.213; Nagelkerke R^2^ = 0.240).

**Table 4:** Multivariate analysis of the factors associated with postoperative DVT following femoral shaft fracture

Variables	OR	95%CI		***P***
		Lower limit	Upper limit	
**Current smoking**	2.902	1.127	7.471	0.027
**Intraoperative blood loss (> 550 ml)**	2.202	1.093	4.436	0.043
**Age (> 35 years)**	2.789	1.197	6.497	0.017
**d**-**dimer (> 1.09 μg/ml)**	4.562	2.257	9.223	< 0.001

## Discussion

The overall morbidity of DVT was comparable with that seen in major orthopedic fractures and appeared not lower than that following hip or knee arthroplasty [7, 16]. Many authors have emphasized the significance of DVT prevention in patients with major fractures, whereas relatively little clinical attention has been paid to patients with femoral shaft fractures. To our best knowledge, this retrospective, case-control analysis was the first large research focused on DVT in patients with surgically treated FSF. Despite adherence to thromboprophylaxis during the hospital stay, 48 of 308 (15.6%) patients developed DVT following FSF surgery. The exact benefit of IPC to DVT prevention remains unclear in this study and varies in different clinical settings, while combined IPC and pharmacological prophylaxis are recommended in patients at high risk of DVT [17]. Different from the previous studies mixed with a spectrum of injuries of the lower extremity including femur fractures, peri-knee fractures, ankle and foot trauma, and even tendon ruptures, this current research specifically involved the FSF subgroup with various potential variables including underlying comorbidities, hematological biomarkers, and operation-related records. Apart from the incidence and characteristics of DVT developed in this population, we also evaluated the independent risk factors that were closely associated with DVT, including current smoking, intraoperative blood loss (> 550 ml), age older than > 35 years, and plasma d-dimer > 1.09 μg/ml.

Although we found no significant link in the univariate analysis between current smoking and postoperative DVT (*p* = 0.095), this association was strengthened with further adjustment for potential confounders in the multivariate logistics regression model, which suggested that smoking was independently associated with DVT in patients undergoing surgery for FSF, with a 2.9-fold elevated risk of DVT in smokers compared with non-smokers. Previous investigations on the relationship between smoking and DVT were inconsistently reported and greatly varied [18, 19], while smoking has been shown to act synergistically with other predisposing factors (e.g., cancer, older age, cardiovascular diseases) in the development of the provoked DVT [20, 21]. In addition, it has been well-established that cigarette smoking was significantly associated with high plasma fibrinogen levels, leading to prolonged coagulation propensity [22, 23]. The risk-increasing impact of smoking may be attributed to multiple pathways or factors it upregulates in the coagulation system, which could be partially explained by the strong relationship between smoking and the presence of DVT in patients sustaining FSF surgery. Although the explicit connection between cigarette smoking and DVT remains unclear, this clinical relevance of the smoking and occurrence of DVT should not be ignored, and the potential benefits of smoking cessation could be underscored during hospital day and post-discharge period.

Many factors were reported as contributors to the formation of thrombosis associated with trauma, including the immobilization and surgical manipulation itself [24]. The univariate analysis suggested the significantly higher blood loss in the DVT group (700.00 ± 471.4 mL), compared to the non-DVTs (495.54 ± 350.1 mL) group. After adopting ROC analysis, the optimum cutoff value specifically related to the subsequent DVT was determined. Our multivariable model result revealed that intraoperative blood loss of more than 550 ml was an independent predictive factor for postoperative DVT (OR = 2.202, *p* = 0.043), regardless of blood transfusion during operation. We hypothesized that blood loss boosted the hypercoagulable states and disruption of the coagulation-fibrinolysis system, which was consistent with the findings by Selby et al. [25] and Riha et al. [26]. Furthermore, the systemic hypercoagulability prone to causing DVT occurred after surgery and persisted to 6 weeks [27], a period longer than the recommended course of thromboprophylaxis use for major orthopedic surgeries. This conclusion could also partially explain another result in the current study that 6 DVTs presented in the bilateral extremities and 5 in the contralateral limb. Up to date, the intrinsic relationship between postoperative DVT and hemostatic changes is still unclear, but the blood loss during operation can be considerably controlled and minimized. By optimizing the surgical procedures and improving the surgeon’s technique, many postoperative complications including DVT might be at a lower incidence.

Advanced age has been well documented to be an independent risk factor of DVT following lower extremity fractures. However, of the previous studies, Pelet et al. [28] failed to identify such relevance in terms of more thromboembolic events and older age, which might be due to that only symptomatic DVTs were examined, and older patients with asymptomatic DVT were likely to be assigned to the non-DVT group in their cohort. Structural and functional alterations of the blood vessels accumulate throughout life, culminating in an increased risk of developing cardiovascular diseases [29]. Although the largest proportion of adult patients with FSF distributes in the young and middle-aged population, the current results revealed that a tendency toward 2.789-fold greater odds of thromboembolic events was noticeable when patients presented with age over 35 years (*p* = 0.017), independent of other variables in this population. A study by Lee et al. [5], comprising nationwide data on patients after major lower limb orthopedic surgery, found that the relative risk of DVT was 5-fold higher in patients aged 50–69 and 10-fold higher in those aged > 70 years compared to those aged < 49 years, as was true in our study. In the setting of trauma, it is essential to remain aware of the structural and functional changes occurring in the vasculature during aging. Timely, appropriate, and effective strategies could be proactively adopted in the advanced age group after early risk stratification.

A prospective observational study revealed some certain fluctuation of endocrinological indexes in patients with surgical treatment of femur fracture [30], while we could hardly find its correlation with DVT given that the routinely tested hematological biomarkers did not include thyroid hormones in the current study. d-dimer is a degradation product originating from fibrinolytic cross-linked fibrin clots and mainly reflects secondary hyperfibrinolysis and thrombosis. With the purpose to exclude DVT, the threshold of d-dimer is clinically set low to maximize the sensitivity and reduce false-negative rates. Several studies reported its cost-effectiveness of thromboembolic disorders combined with or without other screening methods [31, 32]. In a systematic review, Nybo et al. [33] concluded that regardless of the differences in the study design, DVT incidences, and d-dimer assays used, all studies they included were in favor of the age-adjusted d-dimer cutoff with good negative predictive values. In contrast with previous findings, we surprisingly observed the positive relation between DTV prediction and an elevated d-dimer threshold. For this population-adjusted d-dimer value, we found that plasma d-dimer high than 1.09 μg/ml was independently associated with a 4.562 times elevated risk of postoperative DVT in patients undergoing surgery for FSF (*p* < 0.001). Whether the increased utility of d-dimer found in this study was entirely beneficial requires further research, but by far, it seems safe and costs less to build this risk awareness of the population-adjusted d-dimer level in DVT prediction.

From our results, the highlights below are of evidence: firstly, this is a large retrospective study of postoperative DVTs following surgery for closed FSF. Secondly, multiple characteristic data of DVT were clarified in this population, including the incidence, locations in the proximal or distal, unilateral or bilateral, and injured or uninjured. Lastly, the optimal values predicting postoperative DVT were determined from the ROC analyses.

However, there were several limitations related to the retrospective nature of our work. Firstly, the most obvious was the dependence on the quality of the data recorded in the medical records. Furthermore, to improve internal validity, we excluded some patients for data deficiency and other patients with several serious comorbidities (e.g., concurrent fracture in other locations), so our conclusions might not be generalizable to these patients. Additionally, despite modern prophylaxis regimens that would theoretically reduce the odds of a DVT, the current study did not compare the possible differences between individuals. Future prospective, randomized controlled trials should be conducted with additional, longitudinal data.

## Conclusion

In conclusion, we found that 15.6% of patients with isolated FSF manifested DVT within 90 days after surgery. We identified four independent risk factors associated with DVT, including current smoking, intraoperative blood loss (> 550 ml), age older than > 35 years, and postoperative plasma d-dimer > 1.09 μg/ml despite the use of modern thromboprophylaxis. We regard these findings as screening tools to stratify the patients with FSF, make a cross-specialty decision, and implement targeted precaution measures such as quitting smoking and controlling intraoperative blood loss. In addition, our findings would strengthen the evidence regarding the standardized use of thromboprophylaxis for fractures below the hip.

## Data Availability

All the data will be available upon motivated request to the corresponding author of the present paper.

## Abbreviations

DVT: Deep venous thrombosis
FSF: Femoral shaft fracture
DUS: Duplex ultrasonography
LMWH: Low-molecular-weight heparin
IPC: Intermittent pneumonic compression
BMI: Body mass index
ASA: American Society of Anesthesiologists
ORIF: Open reduction internal fixation
OR: Odds ratio
CI: Confidence interval
SD: Standard deviation
